# Nachhaltigkeit in der chirurgischen Niederlassung – ein narratives Review

**DOI:** 10.1007/s00104-022-01785-7

**Published:** 2023-01-05

**Authors:** Nikolaus Christian Simon Mezger, Florian Eickel, Ralph Lorenz, Mirko Griesel

**Affiliations:** 1 Institut für Medizinische Epidemiologie, Biometrie und Informatik, Martin-Luther Universität Halle-Wittenberg, Halle (Saale), Deutschland; 2 Global and Public Health Department, Karolinska Institutet, Stockholm, Schweden; 3 Centre for Planetary Health Policy (CPHP), c/o KLUG – Deutsche Allianz Klimawandel und Gesundheit e. V., Cuvrystr. 1, 10997 Berlin, Deutschland; 4 Praxis Dr. Eickel, Bielefeld, Deutschland; 5 Praxis 3+CHIRURGEN, Berlin, Deutschland; 6 Klinik für Anästhesiologie und Intensivtherapie, Universitätsklinikum Leipzig, Leipzig, Deutschland

**Keywords:** Operationssaal, Klimawandel und Gesundheit, Handlungsempfehlungen, Synergieeffekte, Hürden für Klimaschutz, Operating room, Climate change and health, Recommendations for practice, Synergetic effects, Barriers to climate protection

## Abstract

**Hintergrund:**

Auch die ambulante Chirurgie trägt zur Klimakrise bei. Die Publikation soll die Herausforderungen identifizieren und klare, möglichst evidenzbasierte Empfehlungen für Umweltschutz bei gleichzeitiger Kostenreduktion geben.

**Methode:**

Narratives Review mit nichtsystematischer umfangreicher Recherche in PubMed/MEDLINE und grauer Literatur sowie Befragung von Expert:innen.

**Ergebnisse:**

Eine Vielzahl an Primärarbeiten, Evidenzsynthesen, praktischen Handlungsempfehlungen und Checklisten konnte identifiziert und zwei Expert:innen befragt werden. Umweltprobleme wurden erkannt in Produktion und Beschaffung, Verkehr, beim Verbrauch von Material, Pharmaka und Energie sowie bei Entsorgung, Wiederverwertung und Sterilisation. Hochwertige Publikationen beschreiben nicht einen Mangel an Wissen um Alternativen, sondern an praktischer Umsetzung. Deshalb wurden die Probleme in das 5‑R-Schema („reduce“, „reuse“, „recycle“, „rethink“, „research“) eingeordnet, um Handlungsempfehlungen mit Synergieeffekten bezüglich Kostenreduktion, Patient:innen- und Mitarbeiter:innenzufriedenheit zu präsentieren. Des Weiteren werden Veränderungen der Rahmenbedingungen diskutiert.

**Schlussfolgerung:**

Ambulantes Operieren geht mit relevantem Ressourcenverbrauch einher. Es existieren zahlreiche Möglichkeiten, Umweltschutz mit Kostenreduktion sowie Zufriedenheit von Patient:innen und Mitarbeiter:innen zu verbinden. Für flächendeckenden Klimaschutz in der Niederlassung müssen Anreize und gesetzliche Rahmenbedingungen geschaffen werden.

## Abstract (en)

**Background:**

Surgery is contributing to the climate crisis, not least in the outpatient sector. The present publication aims to identify the challenges this poses, and to provide clear, preferably evidence-based recommendations on environmental protection while simultaneously reducing costs.

**Method:**

Narrative review with a non-systematic search and selection in PubMed/MEDLINE and grey area literature as well as expert interviews.

**Results:**

Numerous primary articles, evidence syntheses, practical recommendations for action and checklists were identified and two experts were interviewed. Environmental issues were identified in the production and procurement, transport of people and goods, usage of materials, pharmaceuticals including anesthetic gases and energy consumption in the outpatient practice and also in disposal, recycling, and sterilization. High-quality publications do not describe a lack of knowledge on alternatives but on a lack of implementation in clinical practice. Therefore, the identified issues were classified in the 5‑R scheme (reduce, reuse, recycle, rethink, research) to present recommendations for action, which are synergetic in terms of cost reduction, patient and staff satisfaction. Furthermore, changes in regulatory frameworks are discussed.

**Conclusion:**

Outpatient surgery comes with relevant consumption of resources and carbon emissions. There are numerous opportunities for action that combine environmental protection with cost reduction as well as patient and staff satisfaction. Incentives, guidelines, and legal framework conditions are needed for comprehensive environmental protection in the private sector.

## Hintergrund

Der Klimawandel ist die größte Gesundheitsbedrohung unserer Zeit, so die Weltgesundheitsorganisation [1]. Menschen im Globalen Süden sind besonders betroffen, [2] aber auch in Deutschland manifestieren sich gesundheitliche Auswirkungen bereits heute, etwa durch eine um 12 % erhöhte Sterblichkeit im Hitzemonat Juli 2022 [3]. Daneben werden rund 70.000 vorzeitige Todesfälle jährlich der Feinstaubbelastung zugerechnet, und sowohl Pollenbelastung als auch vektorübertragene Krankheiten nehmen zu [2]. Vor diesem Hintergrund hat die Gesundheitsminister:innenkonferenz 2020 Beschlüsse für ein nachhaltiges Gesundheitswesen gefasst, [4] und die Bundesärztekammer [5] und Fachgesellschaften [6] rufen dazu auf, Ressourcen und Treibhausgasemissionen einzusparen. Im Vergleich zu Krankenhäusern und der Industrie spielen Arztpraxen eine kleinere Rolle. Dennoch: Operative Disziplinen gehören zu den ressourcenintensivsten im Gesundheitswesen [7]. Gesundheitspersonal kann zudem eine Multiplikatorfunktion für Klimaschutz in der Gesellschaft einnehmen. Diese Übersichtsarbeit zeigt Möglichkeiten auf, in der chirurgischen Praxis Emissionen zu reduzieren. Daneben werden weiterführende Maßnahmen für Praxisteams und Teams in medizinischen Versorgungszentren (MVZ) beleuchtet.

## Methoden

Es liegt bereits ein narratives Review zu Umweltproblemen und der direkten Verknüpfung mit praktischen Handlungsempfehlungen für die Chirurgie vor [8]. Aufbauend auf Recherchen der Autoren zum Klimaschutz in der Praxis [9], dem Handbuch *Grüne Praxen* [10], Checklisten für nachhaltige Praxisführung [11–15] und dem Rahmenwerk *Klimagerechte Gesundheitseinrichtungen* [16] erfolgte mit dem Fokus auf Einsparungen von Emissionen in *CO*_*2*_*-Äquivalenten* (CO_2eq_, im weiteren vereinfacht CO_2_-Emissionen) eine nichtsystematische Literaturrecherche in PubMed/MEDLINE und in Grauer Literatur zu ressourcenschonenden Maßnahmen in der ambulanten Chirurgie. Des Weiteren wurden Expert:innen der Fraunhofer-Einrichtung für Wertstoffkreisläufe und Ressourcenstrategie (IWKS) und vom Netzwerk Zukunft Krankenhaus-Einkauf (ZUKE) konsultiert.

### Im Team ins Gespräch kommen

Bemühungen um CO_2_-Reduktionen haben vor allem Erfolg, wenn alle an einem Strang ziehen. Eine offene Diskussion über praxisinterne Klimaschutzmaßnahmen mit Mitarbeitenden erlaubt es, Potenziale und Reibungspunkte aufzudecken. Welche Maßnahmen wünscht das Team? Welche Maßnahmen können aus welchen Gründen schwer umgesetzt werden? Was macht bestimmte Maßnahmen attraktiv? Effektivität, Machbarkeit und Kontinuität können durch regelmäßige Treffen, Bildung eines Projektteams und/oder eine:n Klimaschutzbeauftragten gestärkt werden.

SMARTe Ziele erleichtern die Umsetzung von Einsparmaßnahmen

#### Beachte.

Ein wesentlicher Schritt ist, vorhandenes Wissen über theoretische Einsparmaßnahmen in tatsächliches Handeln zu übersetzen. Rasche Erfolge stärken die Motivation, alles auf einmal zu wollen ist kontraproduktiv. SMARTe Ziele [17] können dabei eine Hilfestellung sein: S – spezifische, M – messbare, A – attraktive, R – realistische, T – (zeitlich) terminierte Ziele.

### Schwerpunkte setzen

Kohlendioxidemissionen können im Operationssaal bei Materialverbrauch und -entsorgung, in der Energieversorgung, bei Diagnostik und Therapie sowie in der Mobilität von Mitarbeitenden und Patient:innen reduziert werden. Jedes Praxisteam sollte abhängig von Interessen, Neigungen und Erfolgsaussichten individuelle Schwerpunkte setzen (Tab. 1).

**Table 1:** 

**1. Evaluation des Status quo Ihrer Praxis** Am Anfang steht die Nabelschau: Wie klimafreundlich ist Ihre Praxis aufgestellt? Verschaffen Sie sich einen Überblick über die wesentlichen Emissionen. Größere Unternehmen beginnen hier mit einer professionellen Evaluation, die auch eine Berechnung des CO_2_-Fußabdrucks umfasst. Machen Sie es sich aber nicht zu schwer und packen Sie die Sache lieber direkt an
**2. Setzen von (machbaren) Nachhaltigkeitszielen für die Praxis** Wohin soll die Reise gehen? Welche Schwerpunkte sollen gesetzt werden, was könnten erste Schritte sein? Und: Sind alle mit an Bord? Als nächstes sollten Sie sich in der (Gemeinschafts‑)Praxis (erste) Ziele setzen. Findet sich ein Team für die Umsetzung? Bietet es sich an, eine:n Nachhaltigkeitsbeauftragte:n zu ernennen?
**3. Umstellung auf (nachhaltigen) Ökostrom und Ökogas** ❀❀❀ Ein erster und leicht umzusetzender Schritt kann die Umstellung auf nachhaltige Energieversorger sein. Wählen Sie Ihre Anbieter aber mit Bedacht – Angaben wie „Strom aus Wasserkraft“ sind nicht immer gleich nachhaltig
**4. Umstellung auf digitale Dokumentation, nachhaltige Verbrauchsmaterialien, energiesparende IT und Wechsel zu einer nachhaltigen Bank** ❀–❀❀ Die digitale Akte ist in aller Munde – auch aus Nachhaltigkeitsgesichtspunkten. Der Wechsel zu einem nachhaltigen Anbieter für Büromaterialien oder ein Wassersprudler anstatt PET-Flaschen sind relativ einfach umzusetzende Maßnahmen. Erfolgt konsequente Mülltrennung? Das Klimaschutzpotenzial jeder einzelnen Maßnahme ist vergleichsweise gering, dennoch sind die Auswirkungen in den Köpfen der Praxismitarbeitenden mitunter umso größer. Außerdem: Verfolgt Ihre Bank eine nachhaltige Anlagestrategie?
**5. Nachhaltigkeitsmaßnahmen im Operationssaal** ❀–❀❀❀❀❀ Hierfür sind Operationsmanagement und Operationsteam gefragt: Welche Schwerpunkte sehen die Mitarbeitenden? Kann die Anästhesie ins Boot geholt werden? Interne Audits können feststellen, wann der Operationssaal ungenutzt bleibt und welches Energieeinsparpotenzial besteht. Daneben kann das Besteck für Routineoperationen gesichtet werden – welche Instrumente werden entpackt, bleiben aber meistens ungenutzt? Sachgerechte Mülltrennung ist im Operationssaal wegen des wertstofflich wertvollen Chromstahls und der aufwendigen Entsorgung medizinisch-infektiösen Mülls noch wichtiger als im Haushalt. Und: Kann die Anästhesie auf Narkosegase verzichten?
**6. Vermeidung von Pendlerfahrten in privaten Fahrzeugen** ❀❀❀ Dienstfahrrad und Jobticket sind Beispiele für Maßnahmen zur Reduzierung von Pendlerfahrten in privaten Kraftfahrzeugen – und bieten Steuervorteile
**7. Bauliche Veränderungen** ❀❀❀ LED-Leuchten, zentrale Stromschaltung und Zeitschaltuhren bieten ein erhebliches Einsparpotenzial. Wer die Möglichkeit dazu hat, kann über bauliche Veränderungen nachdenken: Verbesserung der Isolierung, Umbau der Heizungsanlage, Photovoltaikanlage etc.
**8. Übers Klima sprechen – mit Patient:innen und Kolleg:innen** ❀–❀❀❀❀❀ Für manche das Schwierigste: Übers Klima reden. Welche Patient:innen sollten vor Hitzewellen geschützt werden? Welche sind empfänglich für Klimaaspekte bei Beratung bezüglich eines gesunden und nachhaltigeren Lebensstils, z. B. in Sachen Ernährung und Bewegung? Und: Wie können Sorgen um z. B. den hohen Materialverbrauch seitens der Chirurgie im Gespräch mit Kolleg:innen enttabuisiert werden? Damit können Sie zur Bewusstseinsbildung um die Dringlichkeit des Handelns beitragen und andere motivieren
**9. (Ehrliche) Kommunikation über die eigenen Nachhaltigkeitsbemühungen** Gutes tun und darüber reden: Kommunizieren Sie Ihre Bemühungen sowohl intern als auch gegenüber Ihren Patient:innen, in Ihrem Netzwerk und in Ihren Stellenanzeigen. Das lädt zum Nachahmen ein. Aber bitte seien Sie dabei schonungslos ehrlich – Greenwashing war gestern

❀ Klimaschutzpotenzial der vorgeschlagenen Maßnahmen

## Einsparbereiche: operative Versorgung

### Materialverbrauch

Für den ambulanten Bereich liegen nur spärliche Daten vor, im Krankenhaus wird der Anteil des Abfalls durch die operativen Fächer auf 20–30 % geschätzt [18]. So ermittelte eine Studie einen Abfall von 7–16 kg pro chirurgischer Behandlung für drei Krankenhäuser in den USA und Großbritannien [19]. Durch Emissionen in der Herstellung und Lieferung von Medizinprodukten entstehen knapp zwei Drittel aller Emissionen des Gesundheitswesens [20]. Demnach können eine Verringerung des Verbrauchs sowie die Wiederverwendung und -verwertung von Medizinprodukten sehr wirksam sein. Bisher werden von Krankenhäusern, Praxen und Herstellern nur vereinzelt kreislaufwirtschaftliche Ansätze verfolgt. Dies beruht auf fehlender Priorisierung, wirtschaftlichen Interessen, Sorge um mangelnde Hygiene, fehlenden Informationen und Zeitmangel, aber auch auf Über- und Fehlregulierung bei der Zulassung von Medizinprodukten und mangelnden Vorgaben des Gesetzgebers, etwa zur verpflichtenden Rücknahme durch die Hersteller [21–23].

#### Merke.

**Figure 1:**
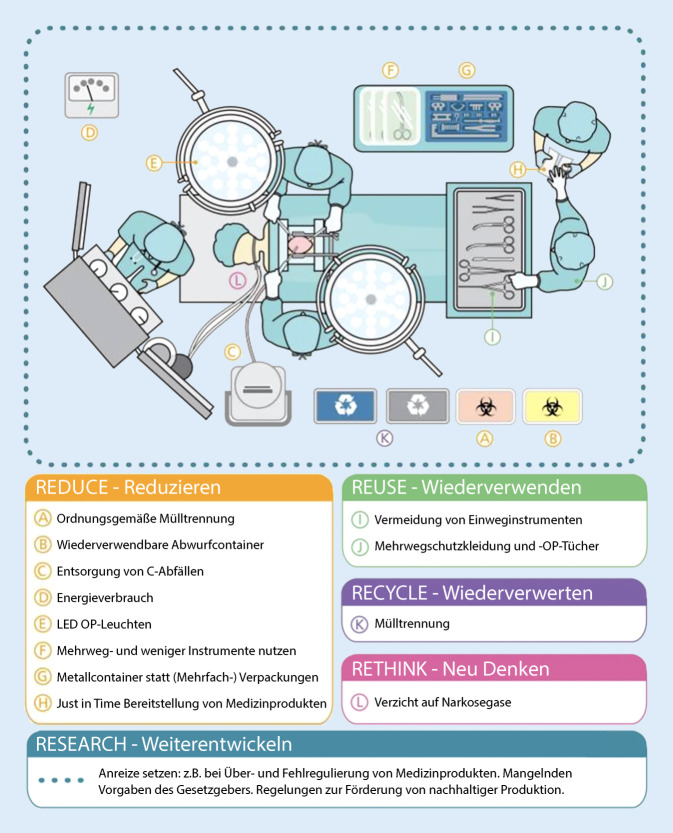

Orientierung gibt das 5‑R-Prinzip: „reduce“ (Reduzieren), „reuse“ (Wiederverwenden), „recycle“ (Wiederverwerten), „rethink“ (Neu denken) und „research“ (Weiterentwickeln, Abb. 1).

#### REDUCE – Reduzierung

Als ein Prinzip der Nachhaltigkeit gilt – weniger ist besser für die Umwelt.

##### Weniger Operationsinstrumente nutzen.

Häufig werden chirurgische Instrumente zwar ausgepackt, jedoch nicht genutzt. In den USA waren dies im neurochirurgischen Bereich 13 % [24], in der Kinderchirurgie sogar 60 % aller ausgepackten Instrumente [25]. Ein kanadisches Krankenhaus zeigte, dass im Hals-Nasen-Ohren-Heilkunde-Bereich nur 36–41 % der standardmäßig entpackten Instrumente tatsächlich verwendet wurden [26]. Insbesondere entpackte endoskopische Instrumente bleiben häufig ungenutzt [27]. Ein bewussterer Einsatz von Instrumenten kann gleichzeitig Umwelt und Finanzen schonen. Operationssets mit stets entpackten und nur bei Bedarf zu öffnenden Instrumenten können im Team entwickelt werden.

##### (Mehrfach‑)Verpackungen vermeiden.

Verpackungsmaterialien tragen erheblich zum Abfallaufkommen bei. Für Mehrweginstrumente können unter Wahrung hygienerechtlicher Vorgaben statt steriler blauer Verpackung (für 19 % des Abfalls im Operationssaal verantwortlich; [18]), wiederverwendbare Metallcontainer genutzt werden, die auch größeren Komfort bieten [28]. Durch größere Bestellvolumina, etwa im Praxenverbund, können zudem Mehrfachverpackungen eingespart werden.

##### Beachte.

Die doppelte Verpackung von Sterilgut ist bei kurzen Transportwegen und geringer Lagerungsdauer verzichtbar [29].

##### Indikationsgerechte Hygienemaßnahmen.

Häufig wird angenommen, die Verwendung unsteriler Handschuhe schütze die Patient:innen vor Übertragung von Infektionen durch das Personal, allerdings dienen sie vor allem dem Eigenschutz. Bei der Arbeit mit offenen Wunden oder Körperflüssigkeit sind zusätzlich zur Händedesinfektion Handschuhe indiziert. Im sonstigen Umgang ist die Desinfektion der Hände des Personals die wesentliche Hygienemaßnahme. Ein englisches Kinderkrankenhaus konnte durch Aufklärung über den Einsatz unsteriler Handschuhe binnen eines Jahres 21 t Abfall einsparen [30].

#### REUSE – Wiederverwenden

##### Mehrweginstrumente.

Die Angst vor Infektionen hat zu vermehrter Verwendung von Einwegmaterialien und damit zu deutlich mehr Abfall geführt [18, 21]. Es gibt jedoch keine hochgradige Evidenz, dass diese Praxis Infektionen verhindert [21]. Im Vergleich von Einweg- und Mehrwegprodukten liegen bisher noch nicht viele Ergebnisse sog. „life cycle analyses“ (systematische Analysen der Energiebilanz von Produkten ausgehend von den Rohmaterialien bis zur Entsorgung/Verwertung) vor, allerdings schneiden Mehrwegprodukte nach den bisherigen Daten insgesamt besser ab [31]. Als Argumente gegen Mehrwegartikel werden häufig finanzieller Druck und hygienerechtliche Bedenken angeführt: Niedergelassene scheuen die vermeintlich kosten-, zeit- und personalintensiven Prozesse bei Reinigung und Sterilisation von Mehrweginstrumenten. Dabei sind Operationsinstrumente zumeist hochwertig (Chrom-/„Chirurgenstahl“) und prädisponiert zur Wiederverwendung. Auch bei manchen elektrisch betriebenen Geräten liegen sterilisierbare Mehrwegalternativen vor, etwa wiederverwendbare Elektrokauter [32]. Entscheiden sich Praxen für Mehrwegprodukte, so können sie sich zur Sterilisierung der Instrumente mit anderen Praxen oder Kliniken zusammenschließen und trotz kurzfristig höheren Anschaffungskosten langfristig Kosten einsparen. Wiederaufbereitung kann auch über externe Anbieter erfolgen, was sich vor allem für städtisch gelegene Praxen und MVZ mit kurzen Transportwegen anbietet. Die Medizinindustrie ist gefordert, ein deutlich größeres und kostengünstigeres Angebot von Mehrwegprodukten zu schaffen [22].

„Remanufacturing“ bedeutet die Wiederaufbereitung von Einweginstrumenten

##### Beachte.

Häufig sind für Einmalverwendung bestimmte Produkte so hochwertig, dass sie wiederverwendet werden können. In den USA hat die Food and Drug Administration (FDA) inzwischen hunderte dieser Einweginstrumente für die Wiederaufbereitung und erneute Nutzung (sog. „remanufacturing“) zugelassen, was die Kosten teilweise halbiert [28]. Bereits 2009 nutzten 25 % aller US-amerikanischen Krankenhäuser und ambulanten operativ tätigen Einrichtungen diese Möglichkeit [14]. Auch in Deutschland gibt es mittlerweile Remanufacturing-Anbieter, etwa für diagnostische und Ablationskatheter und Ultraschallscheren [33]. Hier bietet sich aber noch weit größeres Potenzial [22].

##### Mehrwegschutzkleidung und -operationstücher.

Bei steriler Operationskleidung bergen waschbare Mehrwegalternativen zahlreiche Vorteile. In einer Studie konnten bei 60facher Wiederverwendung von Operationsschutzkleidung im Vergleich zur Einwegvariante eine Verringerung des Verbrauchs von Rohmaterialien um 64 %, Treibhausgasemissionen um 66 %, Wasser um 83 % und Abfall um 84 % erzielt werden [34]. Weitere Analysen zeigten sowohl hinsichtlich der Kosten als auch in Bezug auf Tragekomfort und Schutzqualität Vorteile von wiederverwendbarer gegenüber der im Rahmen der COVID-19(„coronavirus disease 2019“)-Pandemie teurer gewordenen Einwegschutzkleidung [35]. Auch Operationsabdecktücher sind unter ökologischen Vorteilen und ohne erhöhte Infektionsgefahr als reißfeste autoklavierbare Mehrwegtextilien erhältlich [36]. Hier kann die Aufbereitung ebenfalls von externen Partnern übernommen werden [22].

##### Wiederverwendbare Abwurfcontainer.

Zur sicheren Entsorgung spitzer Materialien werden Abwurfbehälter genutzt. Stellen Gesundheitseinrichtungen auf Mehrwegabwurfbehälter um, lassen sich bis zu 84 % der CO_2_-Emissionen einsparen [37].

#### RECYCLE – Wiederverwertung und Recycling

Vielen niedergelassenen Ärzt:innen bereitet der Verpackungsmüll laut einer bundesweiten Befragung Frust und Sorgen, allerdings werden bei Nutzung von Alternativen Mehrausgaben befürchtet [23]. Durch korrekte Mülltrennung kann Geld gespart werden.

##### Wiederverwertung.

Vergleichsweise einfach ist die Abgabe medizinisch-elektrischer Geräte bei zertifizierten Firmen oder direkt beim Hersteller (bei Großgeräten), was aufgrund der gesetzlichen Vorschriften eine Verwertung von mindestens 75 % des Geräts und Wiederverwendung von mindestens 55 % garantiert [38]. Ansonsten ist es bisher schwierig, zur Wiederverwertung beizutragen. Der Verbrauch hochwertiger Chromstahlinstrumente wurde für das deutsche Gesundheitswesen 2020 auf 4000 t geschätzt, die aktuell über Müllheizkraftwerke stofflich entwertet werden. Idealerweise würden diese Operationsinstrumente sterilisiert und wiederverwendet. Wenn dies nicht möglich ist, sollten Chromstahleinweginstrumente getrennt gesammelt, abgeholt und metallurgisch aufbereitet werden [39]. Ähnlich verhält es sich mit Operationsklemmen (Metall- und Kunststoffanteil), die gesammelt und wiederverwertet werden sollten. Hierfür existiert allerdings noch keine flächendeckende und mengenrelevante Infrastruktur [22]. Zur Wiederverwertung weiterer Medizinprodukte mit geringerem Metallanteil und unterschiedlicher Zusammensetzung wird intensiv geforscht.

##### Abfalltrennung.

Operationsabfall besteht überwiegend aus Kunststoff, welcher, sofern nicht mit Körperflüssigkeiten kontaminiert wie Haushaltsmüll im gelben Sack (sog. A‑Abfall) entsorgt und anschließend recycelt oder verbrannt werden kann. Infektiöser Abfall (C-Abfall) erfordert hingegen energie-, ressourcen- und kostenaufwendigen Transport und Vernichtung in einer Sondermüllverbrennungsanlage. Laut den US-amerikanischen Centers for Disease Control and Prevention gelten nur 2–3 % der Krankenhausabfälle als infektiös, jedoch werden bis zu 90 % des Abfalls von Krankenhäusern nicht sachgerecht entsorgt [8]. Weil die Entsorgung infektiösen Abfalls bis zu 5‑mal teurer ist als konventionelle Abfallentsorgung, bietet korrekte Mülltrennung zwischen A‑, B‑ (enthält Bestandteile von Sekreten oder Blut) und C‑Abfall auch und gerade in der ambulanten Chirurgie relevantes Potenzial zur Einsparung von Kosten [14].

Bei der Mülltrennung empfiehlt sich eine enge Zusammenarbeit mit der Anästhesie

##### Wichtig.

Müll sollte sowohl aus Umwelt- als auch aus Kostengründen konsequent getrennt werden. Gerade der beim Entpacken von Sterilgut ohne Kontakt mit Patient:innen entstehende A‑Abfall kann konventionell entsorgt werden. Während des Eingriffs sollte infektiöser Abfall (C-Abfall) von weiterem A‑Abfall (neben Verpackungen z. B. auch Kunststoffinfusionsflaschen) und B‑Abfall (z. B. blut- und sekrethaltige Tupfer) getrennt werden. Bei der Mülltrennung empfiehlt sich enge Zusammenarbeit mit der Anästhesie, deren überwiegend aus A‑Abfall bestehendes Material für 25 % des Operationsabfalls verantwortlich ist [40]. Schulungen und gut sichtbares und leicht verständliches, bebildertes Informationsmaterial können zur Abfalltrennung beitragen.

Die Punkte *RETHINK* (neu denken) und *RESEARCH* (weiterentwickeln) gehen in ihrer Bedeutung über die rein operative Versorgung hinaus und werden somit in den nachfolgenden Kapiteln ausführlich dargestellt.

### Energieversorgung

Operationssäle haben hohe Anforderungen an Luftzirkulation und Temperaturregulierung, nutzen oft energieaufwendige Halogenlampen und haben dadurch einen 3‑ bis 6‑mal so hohen Energieverbrauch wie andere Bereiche von Gesundheitseinrichtungen [31]. In Analysen des CO_2_-Fußabdrucks der Chirurgie spielt die Energieversorgung eine herausragende Rolle – und bietet Einsparmöglichkeiten.

Das größte Potenzial findet sich im Vermeiden von Leerlauf: In den USA bleiben Operationssäle bei laufender Energieversorgung bis zu 40 % ungenutzt [41]. Durch bedarfsangepasstes An- und Abschalten der Energieversorgung von Operationssälen kann auch im ambulanten Bereich gespart werden. Verkürzt sich die Operationszeit und Wartezeit zwischen zwei Eingriffen, hat dies auch positive Effekte auf den Energieverbrauch. Sofern ungenutzte Operationssäle in Betrieb gehalten werden müssen, kann die Luftzirkulation gedrosselt werden, um Energie zu sparen. Daneben können elektrische Geräte und die Beleuchtung bei Nichtnutzung abgeschaltet und Türen zur effizienten Kühlung geschlossen werden, was über Bewegungssensoren automatisiert werden kann [28]. Des Weiteren benötigen LED-Lampen bei vergleichbarer Lichtqualität weniger Strom und erzeugen anders als Halogenlampen kaum Wärme, was wiederum den Stromverbrauch von Klimaanlagen senkt [28]. Beim Neubau von Operationssälen sollte energieeffizientere wasserbasierte Kühlung gegenüber konventionellen Klimaanlagen bevorzugt werden [42].

### Anästhesie

Das Gespräch mit der Anästhesist:in bietet großes Potenzial für den Klimaschutz: Narkosegase sind für 2 % des ökologischen Fußabdrucks des *gesamten *britischen Gesundheitswesens NHS verantwortlich [7], der Anteil im Bereich der operativen Medizin ist entsprechend noch größer. Die Treibhausgaswirkung von Desfluran ist beispielsweise 2000-mal so groß wie jene von CO_2_. Inzwischen empfehlen auch deutsche Fachgesellschaften und das Gesundheitsministerium Baden-Württemberg, auf Desfluran zu verzichten, nach Möglichkeit die total intravenöse Anästhesie (TIVA) zu bevorzugen und zur Wiederverwendung und zum Schutz der Atmosphäre vor Narkosegasen Filtersysteme zu implementieren [6, 43]. Seit kurzer Zeit werden in Deutschland Filter angeboten, die mit geringem Aufwand an viele verfügbare Narkosegeräte nachgerüstet werden können und damit für den ambulanten Bereich besonders interessant sind [44]. Bei geeigneten Patient:innen haben auch regionale/lokale Anästhesieverfahren ökologische Vorteile [11]. Für die klimafreundliche Zusammenarbeit mit Anästhesist:innen liegen zahlreiche Informationsmaterialien vor [6, 45].

Der Verzicht auf Narkosegase verringert wirksam den CO_2_-Fußabdruck

#### Wichtig.

Eine der wirksamsten Maßnahmen zur Verringerung des CO_2_-Fußabdrucks im Operationssaal ist der Verzicht auf Narkosegase, sofern indikationsgerecht. Im britischen Gesundheitswesen NHS (National Health Service) konnte binnen 2 Jahren durch flächendeckende Reduzierung der Nutzung von Desfluran um 55 % eine CO_2_-Ersparnis von 52,9 kt jährlich erzielt werden, was den Emissionen von 192 Mio. Autofahrmeilen entspricht [46]. Ab 2026 darf Desfluran in der EU voraussichtlich nur noch in Einzelfällen genutzt werden [47].

### Überversorgung

Fortschritte der Individualmedizin und stärkere Finanzierung von Gesundheitssystemen haben zwar zu einer Verbesserung der Bevölkerungsgesundheit, aber auch zu einer immer weiter zunehmenden Überversorgung geführt [48]. In Deutschland hat die Initiative *Klug Entscheiden* mit ihren prägnanten 86 Positiv- und 79 Negativempfehlungen Wissen um Vermeidung von Unter- und Überversorgung leicht zugänglich gemacht. Damit sollen unnötige medizinische Leistungen unterlassen und gleichzeitig die Patient:innenversorgung verbessert werden [49]. Bisher liegen zwar keine deutschsprachigen Empfehlungen für chirurgische Fächer vor [50], jedoch sollte evidenzbasierte Medizin auch unter Umweltaspekten Überversorgung und Mehrfachuntersuchungen vermeiden [8].

„Deprescribing“ ist für die Gesundheit von Patient:innen und für die Umwelt förderlich

Des Weiteren sollte der Trend zur Ambulantisierung der Medizin [51] auch als Chance für Umweltschutz gesehen werden: Ohne stationäre Aufnahme werden Ressourcen eingespart – ein Krankenhausbett verbraucht so viel Energie wie vier Einfamilienhäuser [52]. Potenzial für Ambulantisierung chirurgischer Versorgung findet sich in Deutschland etwa bei der Tonsillektomie, Kataraktchirurgie und Teilentfernung der Brustdrüse [51], in der Dermato‑, Hernien‑, Handchirurgie sowie in der Unfallchirurgie und Orthopädie [53–55]. Die Vermeidung einer stationären Aufnahme kann nicht zuletzt auch zum Komfort der Patient*innen beitragen.

#### Beachte.

Die Revision von (Multi‑)Medikation und „deprescribing“ kann nicht nur förderlich für die Gesundheit von Patient:innen, sondern auch der Umwelt zuträglich sein. Die weltweite Herstellung, der Transport und die Nutzung von Medikamenten erzeugen 55 % mehr Emissionen als der weltweite Automobilsektor [56]. Daneben belasten beispielsweise Antibiotika und Diclofenac das Grundwasser [57].

## Einsparbereiche: nichtoperatives Praxismanagement

Auch über den operativen Bereich hinaus gibt es Einsparpotenzial. Einfach umsetzbar sind die Nutzung von Recyclingpapier und die Einführung eines Veggie-Tages im Praxisteam. Verschiedene Checklisten bieten einen umfassenden Überblick über allgemeine Maßnahmen in Praxen und MVZ, so findet sich Inspiration im Handbuch *Grüne Praxen* [10] und auf der Website der Initiative Nachhaltige Praxis der Fachgruppe Health for Future [12]. Saha und Hecker haben praktische Hinweise in der Fachzeitschrift *Deutsches Ärzteblatt* [13] und die Deutsche Allianz Klimawandel und Gesundheit (KLUG) e. V. eine Checkliste [15] veröffentlicht. Darüber hinaus gibt es einen CO_2_-Rechner für Praxen von Wilderness International [58] Im Folgenden sind die Bereiche Energie, Mobilität und nachhaltige Finanzverwaltung zusammengefasst.

### Weitere Energiesparmaßnahmen

Auch jenseits des Operationssaals ist die Energieversorgung eine Stellschraube für mehr Klimaschutz. Einfache Maßnahmen umfassen das Abschalten von Computern, Licht und Ladegeräten nach Dienstschluss, programmierbare Thermostate mit Raumtemperatur von maximal 20 °C und die sachgemäße, energiesparende Einstellung konventioneller Kühlschränke auf 8 °C. Im Winter sollte stoß- statt dauergelüftet werden, im Sommer die Verschattung der Ost- und Südfenster und gezieltes Lüften am Morgen und Abend gegenüber Klimaanlagen bevorzugt werden. Praxen können Strom aus erneuerbaren Energien beziehen. Gerade in MVZ, Gemeinschaftspraxen und Ärztehäusern können in Absprache mit Vermieter:innen bauliche Verbesserungen wie isolierende Fenster, Installation von Photovoltaik und Heizung mittels Wärmepumpen erwogen werden [59].

### Mobilität

Verkehrsmittel rund um den Praxisbetrieb tragen erheblich zu den Emissionen bei, weshalb Patient:innen und Praxisteams (E-)Fahrräder und den öffentlichen Nahverkehr nutzen sollten. Angebote wie Jobtickets, Car-Sharing und Diensträder bieten sogar steuerliche Vorteile und letztere dienen auch der Gesundheitsförderung. Daneben können Fahrgemeinschaften gebildet werden. Die im Rahmen der Pandemie neu geschaffene Möglichkeit von Videosprechstunden kann ebenfalls Emissionen reduzieren und den Patient:innenkomfort durch Zeit- und Kostenersparnis der An- und Abreise erhöhen [59].

### Divestment

Ein weiterer wesentlicher Hebel findet sich im Finanzwesen: Die Verlagerung der Kapitalströme hin zu erneuerbaren Energien wird als entscheidend für die Einhaltung des Pariser Abkommens gesehen [60]. Daher lohnt sich die Überprüfung, Kontaktaufnahme und gegebenenfalls der Wechsel der Hausbank, weil nachhaltige Banken Geld für ethisch und ökologisch vertretbare Zwecke verwenden und nicht in fossile Energien investieren. Bei der Umstellung von Daueraufträgen, Lastschriften und Überweisungen müssen die alte und neue Bank inzwischen helfen.

#### Beachte.

Seit mehreren Jahren wird gesundheitsbezogenes Divestment auch im Gesundheitssektor gefordert [61]. Gemeint ist damit der Abzug von Kapital aus indirekt die Gesundheit schädigenden Industrien (z. B. fossile Energien, industrielle Tierzucht, Waffen, Tabak, Alkohol). Das meiste Kapital im Gesundheitssektor verwalten die privaten Krankenversicherer (> 350 Mrd. €) und die ärztlichen Versorgungswerke (> 100 Mrd. €), die ihre Anlagen überwiegend noch nicht nach transparent nachvollziehbaren Klimaschutzkriterien verwalten [62, 63]. Inzwischen haben manche Versorgungswerke und einzelne Krankenkassen erste Ansätze zur Transformation ihrer Finanzanlagen veröffentlicht [64, 65].

## Weiterführendes Engagement für Nachhaltigkeit

### Klimaanpassung

Häufigere und intensivere Hitzewellen schränken das Wohlbefinden von Patient:innen und auch die Arbeitsfähigkeit von Mitarbeitenden [66] ein. Gegen Hitze in Praxisräumen helfen Verschattung und gezieltes Lüften, Schaffung eines Getränkeangebots, Verzicht auf anstrengende diagnostische und therapeutische Maßnahmen an Hitzetagen und Sprechstunden frühmorgens oder abends für Risikopatient:innen. Kühlen können Ventilatoren, Klimaanlagen sollten sparsam eingesetzt werden, denn sie tragen durch hohen Energieverbrauch zur Klimakrise und durch Abwärme zur Überhitzung der Umgebung bei [67].

#### Weiterlesen.

Weitere Informationen zu Hitzeschutz in der Praxis sind unter: www.hitze.info zu finden.

### Weitere Beratung von Patient:innen

Eine Umfrage der AOK in der Allgemeinbevölkerung zeigt Wissensdefizite zum Zusammenhang zwischen Klimakrise und Gefahren für die menschliche Gesundheit [68]. Ärzt:innen und Medizinische Fachangestellte können hier aufklären und insbesondere Risikogruppen über Schutzmaßnahmen bei Hitzewellen und erhöhter Feinstaubbelastung beraten. Zudem können sie darüber informieren, dass klimafreundliches Verhalten auch gut für die Gesundheit ist: So führt z. B. der Verzicht auf das Auto und mehr Bewegung zu einer Reduktion von orthopädischen Beschwerden, Adipositas oder Depressionen. Durch Fahrradständer und Aushänge mit Fahrzeiten des öffentlichen Nahverkehrs, Loben von Patient:innen, die mit dem Rad kommen, Verweis auf die (unterschätzt [2]) hohe Krankheitslast und Sterblichkeit durch Feinstaubbelastung und die Vorbildfunktion des Praxisteams lässt sich zusätzlich Motivation aufbauen.

Erste Forschungen zur praktischen Durchführung klimasensibler Gesundheitsberatung im ambulanten Bereich zeigen für den deutschen Raum durchaus eine Machbarkeit, wobei die integre Haltung der Ärzt:innen zum Umweltschutz, ihr Wissen zur Klimakrise und der Bezug auf die Gesundheitsprobleme der individuellen Patient:in wesentlich zu sein scheinen [69]. Bundesgesundheitsminister Prof. Lauterbach rief im November 2022 ausdrücklich dazu auf, die Klimakrise im Patient:innenkontakt zu thematisieren und hob die Vorbildfunktion von Ärzt:innen hervor [70].

### Fort- und Weiterbildung des Teams

Eine deutliche Mehrheit niedergelassener Ärzt:innen in Deutschland wünschte sich 2021 mehr Fortbildungen zu Klimawandel und Gesundheit [23]. Erste internationale Evaluationen jüngst entwickelter Kurse zeigen, dass Teilnehmende anschließend mehr Möglichkeiten sehen, im Gesundheitswesen für Nachhaltigkeit und Klimaschutz aktiv zu werden [71]. Mittlerweile bieten einige deutsche Landesärztekammern entsprechende Module an, z. T. auch online. Klimaschutzbeauftragte können Wissen konzentriert weitergeben, hilfreich für den „buy in“ des Praxisteams sind persönliche Narrative zu Klimaschutz und Gesundheit.

#### Weiterlesen.

Für Krankenhäuser gibt es bereits Ausbildungsprogramme zu „Klimamanager:innen“ [72] und zu konkreten Nachhaltigkeitsmaßnahmen im ambulanten Gesundheitswesen ebenfalls vereinzelt Workshopangebote [73, 74].

### Öffentlichkeitsarbeit

Die individuelle Einsparung von Treibhausgasemissionen bleibt ein Tropfen auf den heißen Stein, wenn sie nicht von einer nachhaltigen, sozioökonomischen Transformation flankiert wird. Hier liegt großes Potenzial darin, aus dem Gesundheitssektor heraus Druck für einen gesellschaftlichen Wandel aufzubauen, der ein gutes und gesundes Leben auf einem gesunden Planeten ermöglicht. Mehrere Praxen können sich zu nachhaltig wirtschaftenden Einkaufsgemeinschaften zusammenschließen und bei Herstellern nachhaltigere Produktion, kürzere Lieferketten, ein kostengünstigeres Angebot von Mehrwegprodukten und Nachhaltigkeitssiegel einfordern [22]. Weiterführend können sich Praxisteams im Qualitätszirkel und in Arbeitsgruppen von Fachgesellschaften engagieren. So können politische Lenkungsmaßnahmen wie Gesetze zur zwingenden Rücknahme, Erzielung bestimmter Recycling- und Verwertungsquoten bis hin zu verpflichtenden flächendeckenden Pfandsystemen für Medizinprodukte eingefordert werden [39]. Auf der Homepage können Praxen ihre Bemühungen um Nachhaltigkeit präsentieren sowie Ziele und Erfolge kommunizieren. Dies kann die Attraktivität der Praxis für Patient:innen und auch für potenzielle neue Mitarbeitende erhöhen.

## Unterstützung durch Berufsverbände, kassenärztliche Vereinigungen, Medizinindustrie und Politik

Umfragen unter Ärzt:innen in Deutschland (*n* = 1683, *n* = 514) und Gesundheitspersonal weltweit (*n* = 4654) zeigen eine deutliche Bereitschaft zu Klimaschutz im Gesundheitswesen. Als Barrieren werden mangelndes Wissen und Kenntnisse (*Fähigkeiten*), fehlende Zeit, mangelnde ideelle Unterstützung von Berufsverbänden und Kolleg:innen, erwartete finanzielle Mehrausgaben, ein persönliches Risiko (*Arbeitsbedingungen*) sowie zu geringe erwartete Wirksamkeit von Klimaschutzmaßnahmen und eine erwartete Kontroverse öffentlichen Engagements für Klimaschutz (*Absicht*) genannt [23, 75, 76]. Daraus folgt, dass sich (chirurgische) Berufsverbände und Kassenärztliche Vereinigungen zu Klimaschutz als Aufgabe von Gesundheitsberufen positionieren und auch in Klimaschutzfragen gegenüber Industrie und Politik die Interessen ihrer Mitglieder vertreten sollten. Um die Kluft zwischen Bereitschaft und tatsächlichem nachhaltigem Handeln zu überwinden, sollten Berufsverbände zum einen Gesundheitspersonal mit Informationen und Fortbildungsmöglichkeiten zu Nachhaltigkeitsmaßnahmen *befähigen*. Zum anderen sollten sie mit klarer Positionierung, Agendasetting und Finanzierung, die auch den Zeitmangel in der ambulanten Versorgung berücksichtigen,* entsprechende Bedingungen *für Nachhaltigkeit im Gesundheitswesen schaffen. Zuletzt sollten Untersuchungen zur Wirksamkeit von Klimaschutzmaßnahmen und Verankerung von Nachhaltigkeit in Leitlinien [77, 78] die *Absicht *des Gesundheitspersonals für Klimaschutz stärken. Methoden aus dem Fachgebiet Implementation Science erlauben Monitoring und Adhärenz der Umsetzung.

Nachhaltigere Produktionsbedingungen und Produkte werden dringend benötigt

Zwei Drittel der Emissionen im Gesundheitssektor entstehen in den Lieferketten, also entfernt von den Einrichtungen selbst, und können nur indirekt über die Art der Beschaffung („green procurement“) beeinflusst werden [7, 20]. Gerade im ressourcenintensiven Bereich der Chirurgie werden daher dringend nachhaltigere Produktions- und Transportbedingungen sowie Produkte benötigt, um den hohen Emissionen und der massiven Abfallmenge zu begegnen. Rahmenbedingungen sollten so angepasst werden, dass Klimaschutz und Ressourcenschonung im Gesundheitssektor nicht nur unterstützt werden, sondern der Weg zur Klimaneutralität verpflichtend wird und so Klimaschutz im Gesundheitswesen aus der Nische herauskommt. Denn nur ein klimaneutraler Gesundheitssektor wird den sich bedrohlich verändernden Lebensgrundlagen gerecht [16].

## Schlussfolgerung

Gesunde Ökosysteme sind Voraussetzung für die menschliche Gesundheit. Die Chirurgie trägt in erheblichem Maße zur Umweltverschmutzung bei und ist mitverantwortlich für die sich zuspitzende Klimakrise. Entscheidend ist die Schaffung struktureller Rahmenbedingungen für die rasche und effektive Umsetzung von Klimaschutz: Hierbei sind Berufsverbände, Kassenärztliche Vereinigungen, Medizinindustrie und Politik in der Verantwortung. Es gibt jedoch bereits zahlreiche wirksame Möglichkeiten, in der chirurgischen Niederlassung nachhaltiger zu wirtschaften. Ein Bewusstsein dafür zu schaffen ist ein erster wichtiger Schritt. Für die Umsetzung von Klimaschutz sollten auch und gerade im Kontext knapper zeitlicher und finanzieller Ressourcen alle Mitarbeitenden, aber auch die Patient:innen einbezogen werden. Positive und erfolgreiche Einzelbeispiele sind Multiplikatoren. Tun Sie Gutes und reden Sie darüber, damit die Chirurgie nachhaltig zum Klimaschutz beitragen kann.

## Fazit für die Praxis

- Die Klimakrise bedroht unser aller Gesundheit. Daher ist Klimaschutz auch Gesundheitsschutz – und beginnt im Team. Welchen Wert hat Nachhaltigkeit für Ihr Team und Ihr Unternehmen? Suchen Sie das Gespräch. „Gutes tun und darüber reden“ kann auch die Zufriedenheit von Mitarbeitenden und Patient:innen steigern – aber: Greenwashing war gestern.
- Mit indikationsgerechtem Einsatz von Material, sachgemäßer Entsorgung und gesteigerter Energieeffizienz kann im Operationssaal Nachhaltigkeit gefördert und Geld gespart werden.
- Auf treibhausgaswirksame Narkosegase sollte nach Möglichkeit verzichtet werden. Medikation sollte auch aus Umweltschutzgründen regelmäßig auf ihre Indikation überprüft werden.
- Weiteres Potenzial findet sich in den Bereichen Einkauf, Energie, Mobilität, der Beratung von Patient:innen und Öffentlichkeitsarbeit.
- Noch ist Klimaschutz in der Niederlassung überwiegend Privatsache. Berufsverbände, kassenärztliche Vereinigungen, Industrie und Politik sollten motiviert werden, Nachhaltigkeit im Gesundheitssystem voranzutreiben. Zudem sollten sie mit Informationen, finanziellen Erleichterungen und Vorgaben unterstützen. Die Wirksamkeit aller Maßnahmen sollte evidenzbasiert kontrolliert werden.
