# Erratum zu: Nachhaltigkeit in der chirurgischen Niederlassung – ein narratives Review

**DOI:** 10.1007/s00104-023-01911-z

**Published:** 2023-06-16

**Authors:** Nikolaus Christian Simon Mezger, Florian Eickel, Ralph Lorenz, Mirko Griesel

**Affiliations:** 1 Institut für Medizinische Epidemiologie, Biometrie und Informatik, Martin-Luther Universität Halle-Wittenberg, Halle (Saale), Deutschland; 2 Global and Public Health Department, Karolinska Institutet, Stockholm, Schweden; 3 Centre for Planetary Health Policy (CPHP), c/o KLUG – Deutsche Allianz Klimawandel und Gesundheit e. V., Cuvrystr. 1, 10997 Berlin, Deutschland; 4 Praxis Dr. Eickel, Bielefeld, Deutschland; 5 Praxis 3+CHIRURGEN, Berlin, Deutschland; 6 Klinik für Anästhesiologie und Intensivtherapie, Universitätsklinikum Leipzig, Leipzig, Deutschland

**Erratum zu:**

**Chirurgie 2022**

10.1007/s00104-022-01785-7

In diesem Artikel waren Adressdaten bzw. die Daten zur institutionellen Zugehörigkeit für den Autor Nikolaus Christian Simon Mezger falsch angegeben. Statt „Global and Public Health Department, Karolinska Institutet, Stockholm, Deutschland“ hätte sie „Global and Public Health Department, Karolinska Institutet, Stockholm, Schweden“ lauten sollen. Der Originalbeitrag wurde korrigiert.

